# Safety and effectiveness of sorafenib in Japanese patients with hepatocellular carcinoma in daily medical practice: interim analysis of a prospective postmarketing all-patient surveillance study

**DOI:** 10.1007/s00535-016-1173-5

**Published:** 2016-03-01

**Authors:** Shuichi Kaneko, Kenji Ikeda, Yasushi Matsuzaki, Junji Furuse, Hironobu Minami, Yutaka Okayama, Toshiyuki Sunaya, Yuichiro Ito, Lyo Inuyama, Kiwamu Okita

**Affiliations:** 1 Disease Control and Homeostasis, Kanazawa University Graduate School of Medical Science, 13-1 Takara-machi, Kanazawa, Ishikawa 920-8641 Japan; 2 Department of Hepatology, Toranomon Hospital, Tokyo, Japan; 3 Division of Gastroenterology and Hepatology, Department of Internal Medicine, Tokyo Medical University Ibaraki Medical Center, Ibaraki, Japan; 4 Department of Medical Oncology, Kyorin University School of Medicine, Tokyo, Japan; 5 Division of Medical Oncology/Hematology, Department of Medicine, Kobe University Graduate School of Medicine, Kobe, Hyogo Japan; 6 Pharmacovigilance, Medical Affairs, Bayer Yakuhin, Ltd., Osaka, Japan; 7 Clinical Statistics, Product Development, Bayer Yakuhin, Ltd., Osaka, Japan; 8 Medical Affairs Oncology and Hematology, Bayer Yakuhin, Ltd., Osaka, Japan; 9 Shimonoseki Kosei Hospital, Yamaguchi, Japan

**Keywords:** Hepatocellular carcinoma, Sorafenib, Postmarketing surveillance, Molecular targeted therapy, Japanese

## Abstract

**Background:**

Sorafenib was approved for treatment of unresectable hepatocellular carcinoma (HCC) in Japan in 2009. A prospective postmarketing all-patient surveillance (PMS) study was requested by Japanese authorities to confirm safety and effectiveness of sorafenib in Japanese HCC population.

**Methods:**

Patients with unresectable HCC treated with sorafenib were followed up for 12 months. Data on patient demographic characteristics, treatment status, clinical outcome, and adverse events (AEs) were collected.

**Results:**

This interim analysis included 1109 and 1065 patients evaluable for safety and effectiveness, respectively. Most patients (83.4 %) received the recommended initial dose of 400 mg twice daily. After a follow-up of 12-months, 89.8 % had discontinued treatment, most because of AEs (44.5 %) or progression (33.8 %). The most common drug-related adverse events (DRAE) were hand-foot skin reaction (51.4 %), liver dysfunction (26.4 %), diarrhea (25.1 %), and hypertension (21.6 %). The median overall survival (OS) was 348 days [95 % confidence interval (CI) 299–389 days], and the median duration of treatment was 87 days (95 % CI 78–98 days). Multivariate analyses identified baseline prognostic factors for longer OS, including female sex, low Child-Pugh score, Eastern Cooperative Oncology Group performance status 0, tumor stage I/II/III, low aspartate aminotransferase level, high hemoglobin level, hepatitis C and history of surgical resection.

**Conclusions:**

In general, the safety and effectiveness findings in this PMS were consistent with findings from previous clinical studies. Sorafenib was well tolerated and clinically useful for Japanese patients.

*Clinical trial registration number*: NCT01411436

**Electronic supplementary material:**

The online version of this article (doi:10.1007/s00535-016-1173-5) contains supplementary material, which is available to authorized users.

## Introduction

Hepatocellular carcinoma (HCC) is one of the most common malignancies worldwide [1–3]. The incidence for HCC was 782,000 in the world in 2012 [4] and 43,840 in Japan in 2011 [5]. Although the main etiologic factors for HCC (such as hepatitis B or C virus infection and chronic alcohol abuse leading to the development and progression of cirrhosis) have been identified, about 80 % of HCC cases in Japan have been caused by hepatitis B and C virus infection. Recently, it has been reported that there is a gradual increase of HCC due to non-B non-C hepatitis [6]. At the time of the approval of sorafenib for HCC, patients with advanced HCC or progression after surgical or locoregional therapy had a poor prognosis due to the lack of effective systemic therapy [2, 7, 8]. Cellular signaling that is mediated by the Raf-1 and vascular endothelial growth factor (VEGF) pathways has been implicated in the molecular pathogenesis of HCC [9–12]. Sorafenib tosylate (sorafenib) is an orally active multikinase inhibitor that blocks VEGF receptor (VEGFR) 2 and 3 kinases, and platelet-derived growth factor receptor β (PDGF-β) kinase, as well as Raf-1 kinase, FMS-like tyrosine kinase 3, c-Kit protein, and RET receptor tyrosine kinases [13–15].

In the Sorafenib Hepatocellular Carcinoma Assessment Randomized Protocol (SHARP) study, a phase III multinational, randomized, double-blind, placebo-controlled study, sorafenib significantly prolonged overall survival (OS) and delayed the time to radiologic progression compared with placebo in patients with advanced HCC [16, 17]. Furthermore, a phase III randomized, double-blind, placebo-controlled study in the Asia–Pacific region in patients with advanced HCC demonstrated that sorafenib improved both OS and time to progression [18, 19]. In 2007, sorafenib received an additional marketing authorization with an indication for unresectable HCC in the United States and European Union based on the results of the SHARP study. After the SHARP study, several phase III studies were conducted using molecular targeted drugs [20, 21]. However, no drug other than sorafenib has been approved for advanced HCC.

In Japan, sorafenib received an additional marketing approval for the treatment of unresectable HCC in May 2009 based on the results of a Japanese phase I study [22] and the SHARP study. As a condition of approval, the Ministry of Health, Labour and Welfare in Japan requested implementation of a prospective postmarketing surveillance (PMS) study to confirm the safety and effectiveness of sorafenib in all Japanese patients in the clinical setting.

Information about patient demographic/disease data, treatment status with sorafenib, clinical outcome, tumor response, adverse events (AEs), and other factors potentially affecting safety or effectiveness was collected and is reported here. A part of this paper is based on a third interim report on results of a postmarketing all-patient surveillance study [23].

## Methods

### Study design

This PMS was a prospective, noninterventional study of sorafenib in Japanese patients with unresectable HCC. The aim of this study was to evaluate the safety and effectiveness of sorafenib in a large patient population in daily medical practice in Japan. The primary outcome was to evaluate incidence of adverse drug reactions and serious adverse events, and the second outcome was to assess the effectiveness and confirm the status of therapy with sorafenib. This mandatory PMS registered all patients in advance using a central registration system; patients had to be followed for 12 months after starting treatment, or up to 30 days after discontinuation when it occurred within 12 months.

According to the protocol, an analysis of the PMS was performed for all the patients registered in Japan between May 2009 and January 2010. After 19 January 2010, patient registration continued for all patients, but the follow-up until 14 June 2012 was only for cirrhotic patients with Child-Pugh B or C.

In accordance with the Good Postmarketing Surveillance Practice in Japan, this PMS was initiated in May 2009 in Japan (NCT01411436).

### Patients and treatment

Eligible patients were Japanese patients with unresectable HCC who had been prescribed sorafenib. Patients who had received sorafenib before the registration were included in the safety-analysis set, but not the effectiveness-analysis set. The approved and recommended initial dose of sorafenib was 400 mg orally twice daily.

### Safety assessment

Physical examinations, vital signs, clinical signs and symptoms, and laboratory test results were collected, if available, at visits 1, 3, 6, and 12 months after treatment initiation. A safety analysis was performed in patients who received at least one dose of sorafenib and at least one follow-up visit (safety-analysis set). AEs and drug-related adverse events (DRAE) were coded based on the Medical Dictionary for Regulatory Activities (MedDRA) terminology, version 15.1, and classified as serious or nonserious according to the seriousness criteria defined in International Conference on Harmonization guideline E2A. Clinically similar MedDRA terms were tabulated by combining similar DRAE together.

### Effectiveness assessment

Objective response rate (ORR), disease control rate (DCR), OS (defined as the time from initiation of treatment to death), and the best clinical evaluation were collected. Tumor response was measured according to the clinical evaluation and the Response Evaluation Criteria in Solid Tumors (RECIST) criteria, version 1.0, at the visits for months 1, 3, 6, and 12. The clinical evaluation, an evaluation of comprehensive clinical status by the investigators, was performed based on measures including tumor diameter, clinical manifestation, quality of life, status of primary disease, and tumor marker test results. Best ORR and overall DCR were also calculated, defined as complete response (CR) + partial response (PR) and CR + PR + stable disease, respectively.

### Statistical analysis

In this PMS, point estimates and their 95 % confidence intervals (CIs) were calculated for survival outcome. Survival curves were estimated by the Kaplan–Meier method. Multivariate analyses were performed with exploratory Cox proportional hazards models to assess the association between baseline characteristics and OS, as well as onset time of DRAE. The explanatory variables were initial daily dose, sex, age, weight, Child-Pugh score, Eastern Cooperative Oncology Group performance status (ECOG PS), tumor stage, baseline laboratory test [aspartate aminotransferase (AST), alanine aminotransferase, platelet count, hemoglobin (Hb) and creatinine], etiology (hepatitis B, hepatitis C and alcohol abuse), liver cirrhosis, previous interferon use, sites of extrahepatic metastases (lymph nodes, lung, and bone), comorbidity (cardiovascular disease, hypertension, diabetes mellitus, and high risk for hemorrhage), previous therapy [surgical resection, hepatic arterial infusion chemotherapy (HAIC), systemic chemotherapy, percutaneous ethanol injection therapy (PEIT), radiofrequency ablation, transcatheter arterial embolization/chemoembolization (TACE), and radiotherapy]. With regard to laboratory parameters, the median was selected as the cutoff.

All the variables were to be evaluated in principle, but the variables missing in more than 10 % of the patients or showing extreme distribution, which was biased to one of the levels in more than 95 % of the patients, were excluded. All statistical analyses were performed using SAS version 9.1 or higher (SAS Institute Inc., Cary, NC, USA). For all statistical testing, given the exploratory nature of the study, a nominal *p* value <0.05 (two-sided) was considered significant, and no adjustment for multiple testing was made.

Safety data were summarized with descriptive statistics. The onset time of DRAE was evaluated by calculating the ratio of cumulative incidence rate at day 30 to that at month 12 using the Kaplan–Meier method. To assess the relationship between DRAE and survival outcome, exploratory landmark analysis was conducted to minimize the confounding bias (long-term survivors have a greater chance of DRAE). Patients who survived more than 30 days after the start of treatment were stratified by the presence or absence of specified DRAE (DRAE or DRAE groups with ≥5 % incidence) on day 30, and survival thereafter was compared by Kaplan–Meier analysis.

## Results

### Patients

There were 1119 patients enrolled from 462 investigation sites/departments between May 2009 and January 2010. Ten patients were excluded from the safety-analysis set (*n* = 1109), five for whom the case report form was not collected, two who failed to return after the first visit, and three who transferred to another hospital. The effectiveness-analysis set included 1065 patients after excluding 44 patients who had been treated with sorafenib before enrollment.

Among baseline characteristics of the patients, hepatitis C (52.6 %) was the most common etiology of HCC, followed by hepatitis B (24.1 %), alcohol abuse (9.4 %), and nonalcoholic steatohepatitis (2.2 %). Patients with tumor stage IV (TNM classification based on the criteria of the Liver Cancer Study Group of Japan) comprised more than 70 % (stage IV A/B, 26.2 %/46.9 %), and most patients (97.3 %) had good performance status (ECOG PS score 0 or 1). Although 10.6 % of the patients in Child-Pugh class B and 0.2 % of Child-Pugh class C were enrolled, the majority of patients were Child-Pugh class A (88.0 %). Most patients had previous therapy (91.9 %; Table 1). At 12 months, 113 patients (10.2 %) remained on treatment and 996 (89.8 %) discontinued, most because of AEs (44.5 %) or progression (33.8 %). The majority of patients (83.4 %) received 800 mg as initial daily dose, and 599 (54.0 %) experienced dose interruption and/or reduction; as a result, the median average daily dose was 614.3 mg, and the median duration of treatment (DOT) was 87.0 days (Table 2).

**Table 1:** Baseline demographic and disease characteristics of patients (safety-analysis set)

Variable	Overall (*n* = 1109)	Variable	Overall (*n* = 1109)
Sex *n* (%)		Child-Pugh status *n* (%)	
Male	892 (80.4)	A	976 (88.0)
Female	217 (19.6)	B	118 (10.6)
Age, years		C	2 (0.2)
Median (range)	69.0 (12–90)	Child-Pugh score *n* (%)	
Etiology^a^ *n* (%)		5	510 (46.0)
Hepatitis B	267 (24.1)	6	466 (42.0)
Hepatitis C	583 (52.6)	7	82 (7.4)
Alcohol abuse	104 (9.4)	8	30 (2.7)
Nonalcoholic steatohepatitis	24 (2.2)	9	6 (0.5)
Tumor stage (TNM classification)^b^ *n* (%)		10	0
		11	2 (0.2)
I	10 (0.9)	Previous therapy^a^ *n* (%)	
II	49 (4.4)	Yes	1019 (91.9)
III	227 (20.5)	Surgical resection	423 (38.1)
IV A	291 (26.2)	Locoregional therapy	862 (77.7)
IV B	520 (46.9)	Transarterial chemoembolization	755 (68.1)
Extrahepatic metastases *n* (%)			
Present	598 (53.9)	Percutaneous ethanol injection	162 (14.6)
In lymph nodes	177 (16.0)		
In lungs	328 (29.6)	Radiofrequency ablation	387 (34.9)
Cirrhosis *n* (%)			
Present	691 (62.3)	Radiotherapy	118 (10.6)
Comorbidity *n* (%)		Hepatic arterial infusion chemotherapy	365 (32.9)
Present	880 (79.4)		
Hypertension	483 (43.6)	Systemic chemotherapy	324 (29.2)
Diabetes mellitus	296 (26.7)	AFP (ng/mL)	
ECOG PS score *n* (%)		Median (range)	282.0
0	778 (70.2)		(0–1,804,000.0)
1	300 (27.1)		
2	20 (1.8)		
3	10 (0.9)		
4	1 (0.1)		

*TMN* tumor, node and metastasis, *ECOG PS* Eastern Cooperative Oncology Group performance status, *AFP* α-fetoprotein

^a^A subject could have more than one etiology or previous therapy

^b^TNM classification based on the criteria of the Liver Cancer Study Group of Japan

**Table 2:** Status of treatment continuation/discontinuation and drug exposure (safety-analysis set)

	Whole period (*n* = 1109)	
Initial daily dose		
<800 mg	184	(16.6)
800 mg	925	(83.4)
Treatment status		
Continuation after 12 months	113	(10.2)
Discontinuation	996	(89.8)
Due to		
AE	494^a^	(44.5)
Progression	375^a^	(33.8)
Other/unknown reasons	248^a^	(22.4)
Dose interruption/reduction		
Interruption	350	(31.6)
Reduction	374	(33.7)
Interruption/reduction	599	(54.0)
Median daily dose, mg/day (range)	614.3	(84–800)
Median duration of treatment, days (95 % CI)	87.0	(78–98)

Values represent the number (%) of patients

*AE* adverse event, *n* number of patients evaluated, *CI* confidence interval

^a^A subject could have discontinued owing to more than one reason

### Safety

During the observation period, 90.2 % of patients experienced at least one DRAE. The most common DRAE were hand-foot skin reaction (51.4 %), liver dysfunction (26.4 %), diarrhea (25.1 %), hypertension (21.6 %), rash (16.1 %), and anorexia (15.0 %). Several serious DRAE were also observed, with liver dysfunction (12.9 %) being the most frequent (Table 3). Hand-foot skin reaction (6.3 %), liver dysfunction (5.8 %), diarrhea (4.9 %), and anorexia (4.6 %) were the most frequent AEs leading to discontinuation (data not shown).

**Table 3:** DRAE occurring in ≥ 2 % of patients (safety-analysis set)

	All, *n* (%)	Serious, *n* (%)
Number of patients with DRAE	1000 (90.2)	391 (35.3)
Hand-foot skin reaction^a^	570 (51.4)	36 (3.2)
Liver dysfunction^a^	293 (26.4)	143 (12.9)
Diarrhea	278 (25.1)	20 (1.8)
Hypertension^a^	239 (21.6)	4 (0.4)
Rash	178 (16.1)	12 (1.1)
Anorexia	166 (15.0)	27 (2.4)
Alopecia	116 (10.5)	0
Platelet count decreased	106 (9.6)	23 (2.1)
Increase in lipase/amylase^a^	99 (8.9)	1 (0.1)
Pyrexia	83 (7.5)	16 (1.4)
Malaise	69 (6.2)	9 (0.8)
Ascites	56 (5.0)	16 (1.4)
Gastrointestinal bleeding^a^	53 (4.8)	51 (4.6)
Nausea/Vomiting^a^	51 (4.6)	3 (0.3)
Stomatitis	44 (4.0)	0
Dysphonia	40 (3.6)	0
Fatigue	38 (3.4)	2 (0.2)
Hypophosphataemia	34 (3.1)	4 (0.4)
Anemia	28 (2.5)	14 (1.3)
Erythema multiforme	24 (2.2)	24 (2.2)

The values represent the number of patients

*DRAE* drug-related adverse events, *MedDRA* Medical Dictionary for Regulatory Activities

^a^Clinically similar terms of MedDRA were combined in one DRAE

**Fig. 1:**
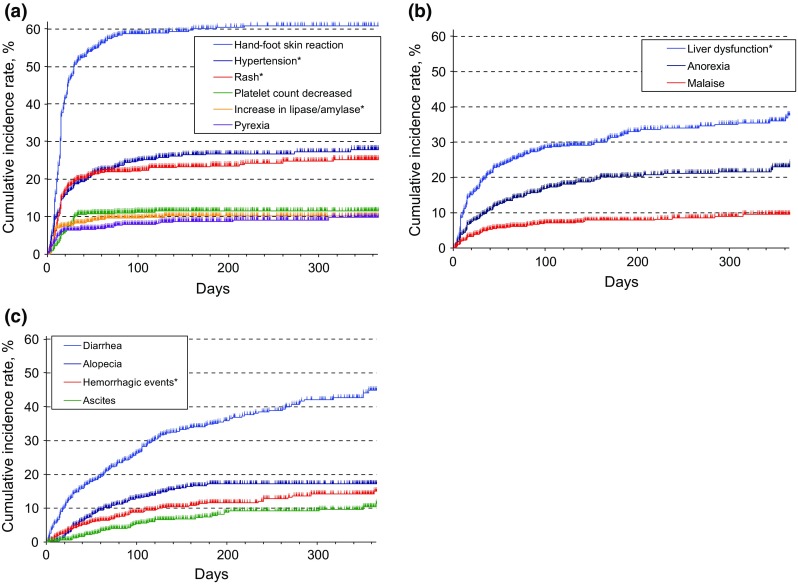
Time course of onset of drug-related adverse events (DRAE) evaluated based on the ratio of cumulative incidence rate at day 30 to that at day 365 using the Kaplan–Meier method: **a** DRAE occurring in the early stage of treatment (ratio ≥ 0.6); **b** intermediate DRAE (ratio ≥0.4 to <0.6); **c** DRAE occurring over the entire period (ratio <0.4). *Clinically similar terms of MedDRA were combined in one DRAE. *MedDRA* Medical Dictionary for Regulatory Activities

The time course of onset of DRAE is shown in Fig. 1. DRAE, including hand-foot skin reaction, hypertension, rash, platelet count decreased, increased lipase/amylase, and pyrexia, occurred predominantly in the early stage of treatment [ratio of incidence rates (day 30/day 365), ≥ 0.6], whereas diarrhea, alopecia, hemorrhagic events and ascites occurred over the entire period (ratio < 0.4). liver dysfunction, anorexia, and malaise were intermediate (ratio ≥ 0.4 to < 0.6).

In total, 39 patients (3.5 %) died from DRAE (54 events), including liver dysfunction (six events), hepatic encephalopathy, hepatic failure, and interstitial lung disease (three events each) (data not shown). Twenty-nine patients died from other causes in addition to the drug-related cause, and 19 of the 29 patients died from the primary disease-related causes in addition to the drug-related cause.

Multivariate analyses using Cox proportional hazard models identified baseline characteristics that were associated with increased risk of DRAE. The variables were selected with the stepwise method. Initial daily dose of 800 mg, female sex, ECOG PS score of 0, AST level below the median value (51 IU/L), Hb level at or above the median value (11.8 g/dL), previous surgical resection, and PEIT were risk factors for hand-foot skin reaction. Patients with ECOG PS score of 1, AST level above the median value, comorbidity with high risk for hemorrhage, or previous HAIC had an increased risk of serious liver dysfunction (Table 4).

**Table 4:** Multivariate analysis of baseline risk factors associated with the development of hand-foot skin reaction, serious liver dysfunction and baseline variables

Drug-related adverse events	Variable		Incidence Rate^a^ (/person-years) (*n* = 922)	Hazard ratio	95 % CI	*p* Value
Hand-foot skin reaction	Initial daily dose	800 mg	3.7	1		
		<800 mg	1.7	0.54	0.41–0.71	<0.0001
	Sex	Male	3.1	1		
		Female	4.0	1.46	1.16–1.83	0.0011
	ECOG PS	0	3.5	1		
		≥2	1.7	0.46	0.22–0.97	0.0406
	AST (51 IU/L^b^)	<Median value	3.3	1		
		≥Median value	3.4	0.80	0.67–0.96	0.0187
	Hemoglobin (11.8 g/dL^b^)	<Median value	2.7	1		
		≥Median value	3.8	1.42	1.18–1.71	0.0002
	Previous surgical resection	Absent	2.9	1		
		Present	3.8	1.22	1.01–1.46	0.0362
	Previous PEIT	Absent	3.2	1		
		Present	3.6	1.30	1.03–1.64	0.0303
Serious liver dysfunction	ECOG PS	0	0.3	1		
		1	0.6	1.49	1.02–2.18	0.0412
	AST (51 IU/L^b^)	<Median value	0.2	1		
		≥Median value	0.6	2.72	1.85–3.99	<0.0001
	Comorbidity with high risk for hemorrhage	Absent	0.4	1		
		Present	0.5	1.60	1.02–2.52	0.0408
	Previous HAIC	Absent	0.3	1		
		Present	0.6	1.63	1.13–2.34	0.0089

Among the baseline variables tested, only the variables for which a significant association was detected were presented

*n* number of patients evaluated, *CI* confidence interval, *ECOG PS* Eastern Cooperative Oncology Group performance status, *AST* aspartate aminotransferase, *PEIT* percutaneous ethanol injection therapy, *HAIC* hepatic arterial infusion chemotherapy

^a^Evaluated 922 patients (83.1 %) available for this analysis in the safety-analysis set of 1109 patients

^b^Median value

### Outcome

**Fig. 2:**
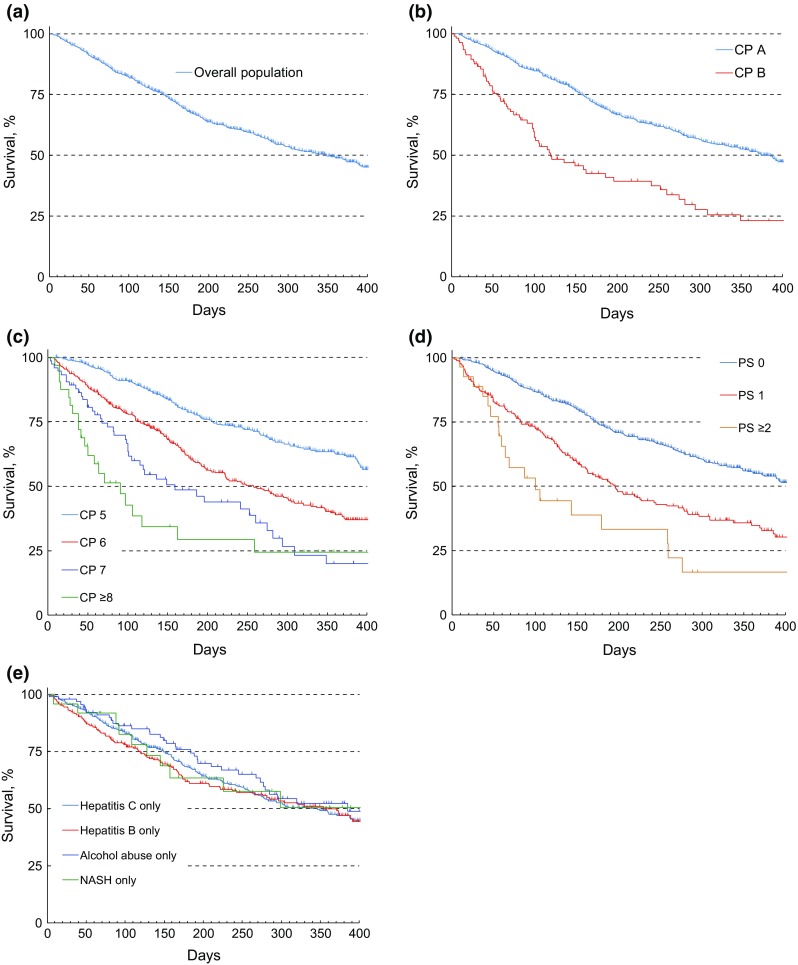
Kaplan–Meier analyses of OS (effectiveness-analysis set): **a** OS of overall population of patients; **b** OS by baseline Child-Pugh class (CP A and CP B); **c** OS by baseline Child-Pugh score (A5, A6, B7, and ≥B8); **d** OS by baseline ECOG PS (PS 0, 1, and ≥2); and **e** OS by etiology. *OS* overall survival, *CP* Child-Pugh, *ECOG PS* Eastern Cooperative Oncology Group performance status, *NASH* nonalcoholic steatohepatitis

The ORR was 5.4 %, and DCR was 39.3 % (Table S1), and the median OS was 348 days (95 % CI 299–389 days). The median OS decreased from Child-Pugh A5 [452 (95 % CI 404–519) days], A6 [258 (210–302) days], B7 [159 (101–260) days], to ≥ B8 [91 (41–162) days; Fig. 2].

**Fig. 3:**
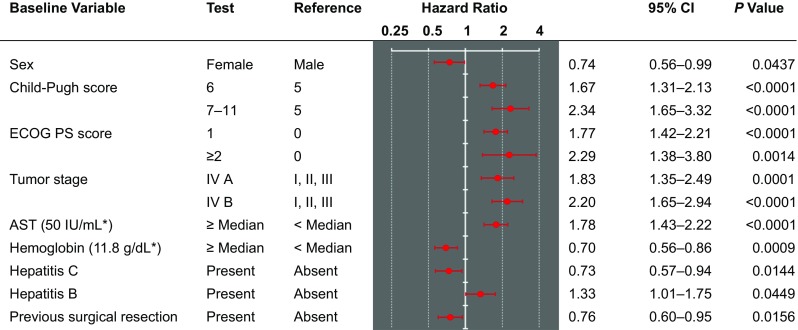
Overall survival of patient subgroups according to baseline prognostic factors. Evaluated 887 patients (83.3 %) available for this analysis in the effectiveness-analysis set of 1065 patients. *Median value. *CI* confidence interval, *ECOG PS* Eastern Cooperative Oncology Group performance status, *AST* aspartate aminotransferase

The multivariate analyses evaluated 887 patients available in the effectiveness-analysis set and identified baseline characteristics that were correlated with longer OS: female sex, Child-Pugh A5, ECOG PS score of 0, tumor stage I/II/III, AST level (below median) and Hb (at or above median), hepatitis C, and history of surgical resection (Fig. 3).

To elucidate an association between DRAE and OS, landmark analysis was performed. OS was statistically longer for patients with hand-foot skin reaction or hypertension within 30 days: hand-foot skin reaction (1-year OS with and without DRAE, 59.5 vs. 42.8 %, *p* < 0.0001 by log-rank test), hypertension (59.4 vs. 49.2 %, *p* = 0.0195; Fig. S1).

## Discussion

This report shows the results of a prospective, large-scale surveillance study in which data for all patients treated with sorafenib were collected following the approval of sorafenib for the treatment of HCC in Japan.

In this analysis involving 1109 Japanese patients with unresectable HCC on sorafenib treatment in real-life clinical practice, the most common DRAE were hand-foot skin reaction (51.4 %), liver dysfunction (26.4 %), diarrhea (25.1 %), hypertension (21.6 %), rash (16.1 %), and anorexia (15.0 %); the majority of safety data obtained were broadly comparable to those reported in previous clinical studies [16–19], although the incidences of some DRAE such as hand-foot skin reaction, liver dysfunction, and hypertension were higher compared with those in the previous studies. Apart from this PMS, the Global Investigation of therapeutic DEcisions in hepatocellular carcinoma and Of its treatment with sorafeNib (GIDEON), a global, prospective, noninterventional study investigating the safety of sorafenib in unresectable HCC patients in real-life practice, was started after this PMS, and 517 patients were enrolled from Japan. Based on these data, the frequency of most common DRAE in the PMS reported here is similar to that reported for Japanese HCC patients in GIDEON (data not shown). In real-life clinical practice, unexpected safety information was not identified, and most DRAE were manageable.

When the timing of DRAE onset was analyzed, the results showed that hand-foot skin reaction, hypertension, and rash developed relatively early after initiation of treatment with sorafenib, whereas diarrhea occurred over the entire period and was continuously reported after the initiation of the treatment. The incidence pattern of DRAE in the present study is similar to that shown in patients with radical unresectable or metastatic renal cell carcinoma in a prospective postmarketing all-patient surveillance study (*n* = 3255), conducted in Japan [24]. Since patients tended to experience DRAE at a different timing depending on the type, DRAE need to be managed based on their characteristics.

The multivariate analysis revealed that female sex and an ECOG PS score of 0 were risk factors for hand-foot skin reaction. Previous studies have shown that female sex and ECOG PS score of 0 are risk factors for hand-foot skin reaction, indicating that our results are consistent with the previous studies [24, 25]. Hand-foot skin reaction is one of the frequently found DRAEs. Although hand-foot skin reaction is not directly life-threatening, it worsens patient’s quality of life and is a major cause of cessation of treatment with sorafenib. However, since hand-foot skin reaction could be prevented by care of limbs, and application of steroids and urea cream, an appropriate measure is important even before the initiation of treatment with sorafenib [26, 27]. Although median DOT was 15.9 weeks in GIDEON in the Japanese subpopulation, it was 87.0 days (12.4 weeks) in this PMS. There are a number of factors that may underlie the difference in median DOT between GIDEON and this PMS. A ratio of patients with stage IV (TNM classification based on the criteria of the Liver Cancer Study Group of Japan) before initiation of treatment with sorafenib was 73.1 % in this PMS; whereas that in GIDEON was 44.3 %, suggesting that the worse patients’ baseline characteristics in this PMS are associated with the shortened DOT (data not shown). In addition, the PMS was performed in all institutions that administered sorafenib in Japan, while 40 Japanese institutions with considerable experience of the administration of sorafenib participated in GIDEON. Furthermore, when the DOTs in Japan and the other regions reported in the GIDEON study were compared, the results showed that the DOTs tended to be shorter in the Asia Pacific region (12.6 weeks) and the USA (12.7 weeks), and longer in the European Union (17.1 weeks) and Latin America (23.1 weeks) (data not shown). Two randomized phase III studies showed that sorafenib therapy obtained survival benefit over placebo and recommended initial dose of sorafenib of 800 mg/day. On the other hand, initial dose setting is controversial because risk factors required for reduced dose for those who are 80 years or older, those who had a body weight of 40 kg or less, those with poor renal function, and those who had a history of treatment for varices or ascites are reported [28]. In addition, it is also reported that unresectable HCC patients treated with initial half-dose sorafenib treatment had comparable OS and TTP compared with those treated with initial standard dose sorafenib [29, 30]. Therefore, prospective, randomized clinical trials are necessary to clarify initial dose setting. Multicenter, Randomized Pilot Study of the Effect of Sorafenib Dosing Schedule on Tolerability and Drug Delivery (NCT01203787) is being conducted. SOFIA study reported that properly managing DRAE and modifying the dose after initiation of 800 mg/day is important for obtaining clinical benefit [31]. Taken together, it is critical to extend the DOT by modifying the dose after initiation of sorafenib therapy and properly managing the DRAE in compliance with proper use guide to fully receive the benefit of treatment with sorafenib. Especially with regard to hand-foot skin reaction, appropriate management is critical because it is reported that the development of hand-foot skin reaction within 4 weeks after sorafenib initiation is closely associated with longer OS [32].

The median OS in this study was 348 days, which is in line with OS in the previously reported studies. The results showed that the survival curve was separated depending on the Child-Pugh score. The results of this study also showed that patients with Child-Pugh class B tended to have short survival duration. Therefore, paying due consideration to a balance between the benefits and risks, a treatment strategy including other therapies should be considered in patients with Child-Pugh class B. However, since the GIDEON study has shown that the baseline albumin and bilirubin values are critical for prognostic factors for OS, not predictive factor for treatment effects, it appears that there is also a group of patients with Child-Pugh class B who can receive some benefit [33]. Some baseline characteristics that affect OS prolongation were identified by the multivariate analysis. Although most of those characteristics are previously reported prognosis factors, hepatitis C virus—positive status was determined as an independent prognosis factor in this study. Japanese retrospective study has also revealed that seven factors (distant metastases, portal invasion, intrahepatic tumor burden, serum AFP, DCP, albumin and total bilirubin) were independently related to a worse survival [34]. Although the same prognostic factors were not identified, factors related to liver function were common in both studies. Therefore, it is suggested that maintaining the liver function during treatment is important. As for the predictive factors of sorafenib, sorafenib showed better OS in patients with hepatitis C virus–positive status than other drugs in exploratory analyses of previous studies [20, 21]. In addition, the SHARP/AP pooled analysis showed that hepatitis C virus-positive status was identified as a predictive factor of sorafenib. As the prognostic factors are not the same as the predictive factors, it is important to distinguish between the two factors correctly [35].

The results of the exploratory landmark analysis indicated that longer OS is associated with early-onset DRAE (hand-foot skin reaction and hypertension) within 30 days. Reig et al. have prospectively investigated the therapeutic-effect-related factors in advanced hepatocellular carcinoma, based on the patients’ background including AEs, and reported that the expression of skin-related events within 60 days after the initiation of treatment with sorafenib is associated with a better OS [36].

Therefore, it is important to manage these DRAE from the beginning of treatment so that the number of discontinued cases due to the early-onset DRAE can be decreased, and a longer treatment can be achieved.

An AST level that is higher than median level before treatment and previous HAIC were also identified as risk factors for serious liver dysfunction by the multivariate analysis. Therefore, patients with such characteristics should be treated with caution to reduce the risk of developing hepatic disorder during treatment with sorafenib. HAIC is usually performed for advanced HCC not indicated for surgical resection or TACE. Patients with previous HAIC may have diminished hepatic functional reserve. In addition, it was considered that sorafenib was administered at a later line in patients with previous HAIC, which was further in the progression of the disease. The analysis of deaths developed relatively early (within 30 days after the initiation of the treatment), regardless of causal relationship with sorafenib, was performed after the launch of sorafenib in Japan; it showed that 18 % of deaths had an AST level > 200 IU/L before initiation of treatment with sorafenib. Rapid increase or transaminase levels > 200 IU/L after initiation of treatment were also associated with death. Based on these results, the proper use advisory committee for this drug strongly recommends avoiding treatment with sorafenib in patients with a transaminase level > 200 IU/L and careful monitoring of transaminase levels after initiation of the treatment. It is necessary to pay attention to the liver dysfunction because a high incidence of liver dysfunction has also been reported in studies other than this study [32, 37, 38]. The liver dysfunction may be managed by carrying out appropriate measures, such as observation and tests with a frequency of once a week during the first month after the initiation of treatment with sorafenib, as recommended by the proper use advisory committee for sorafenib. An incidence of serious liver dysfunction has been declining since the alert in December 2009 (data not shown). This PMS study is thought to include the data similar to those obtained from the general population, because the study included the data obtained from all the cases during the predetermined term. However, it is possible that there was a selection bias, because the present study was not designed as a randomized controlled study. Furthermore, as source data verification was not carried out, there may be some discrepancy in the data between the source document and case report form.

However, this study provides healthcare professionals with valuable data regarding the safety and usefulness of sorafenib for HCC based on real-world data.

In conclusion, there was no new safety concern and the safety profiles of DRAE were in line with those in previous studies. OS in patients treated with sorafenib was in line with that in the registration Phase III study. Sorafenib was well tolerated and clinically useful for Japanese patients.

## Electronic supplementary material

Below is the link to the electronic supplementary material.

Supplementary material 1 (DOC 182 kb)
