# Supplementary material for: Safety and effectiveness of sorafenib in Japanese patients with hepatocellular carcinoma in daily medical practice: interim analysis of a prospective postmarketing all-patient surveillance study
Source: J Gastroenterol. 2016 Mar 1;51(10):1011–21. doi: 10.1007/s00535-016-1173-5 (PMC5037148; doi:10.1007/s00535-016-1173-5)
Supplement: Supplementary file 1 — Supplementary material 1 (DOC 182 kb) [file 535_2016_1173_MOESM1_ESM.doc]

| (a) | (b) |
| --- | --- |
|  |  |

**Fig. S1**

**Table S1.** Summary of effectiveness measures assessed based on the clinical evaluation and the Response Evaluation Criteria in Solid Tumors criteria (effectiveness-analysis set)

| Effectiveness measure | Overall |
| --- | --- |
|  | (*n* = 1065) |
| Best clinical evaluation |  |
| Effective | 155 (14.6) |
| No change | 331 (31.1) |
| Progression | 392 (36.8) |
| Indeterminate (unevaluable/unknown) | 187 (17.6) |
| Best overall response by the RECIST criteria |  |
| Complete response | 8 (0.8) |
| Partial response | 49 (4.6) |
| Stable disease | 362 (34.0) |
| Progressive disease | 353 (33.1) |
| Indeterminate (unevaluable/unknown) | 293 (27.5) |
| Objective response rate (%) | 5.4 |
| Disease control rate (%) | 39.3 |

Values represent the number (%) of patients

*n* number of patients evaluated, *RECIST* Response Evaluation Criteria in Solid Tumors
